# Revisiting cost-effectiveness of folic acid supplementation in primary stroke prevention in China: considering vitamin B12 deficiency masking issue

**DOI:** 10.1186/s12889-024-21005-7

**Published:** 2024-12-19

**Authors:** Xiyin Chen, David Bishai

**Affiliations:** 1 School of Public Health, LKS Faculty of Medicine, The University of Hong Kong, Hong Kong SAR, China; 2 Department of International Health, Johns Hopkins Bloomberg School of Public Health, Baltimore, MD USA

**Keywords:** Cost-effectiveness analysis, Decision science, Folic acid supplementation, Vitamin B12 deficiency screening, Primary stroke prevention, China

## Abstract

**Objectives:**

To identify the cost-effectiveness of four policy options related to folic acid supplements after considering the side effects of masking vitamin B12 (B12) deficiency in primary stroke prevention for hypertensive patients in China.

**Study Design:**

A cost**-**effectiveness analysis.

**Methods:**

Four policies were considered: Policy A, Do nothing to address folate status in hypertensive patients at risk for stroke; Policy B, Folate supplementation without pre-screening for vitamin B12 deficiency; Policy C, Folate supplementation with pre-screening all patients for B12 deficiency and add B12 supplements if B12 is deficient; and Policy D, Folate supplementation only for those whose folate is deficient, pre-screen all patients for both B12 and folate deficiencies and add B12 supplements if B12 is deficient. A decision tree with a five-year period of intervention based on the China Stroke Primary Prevention Trial (CSPPT) from the Chinese healthcare system perspective estimated incremental cost-effectiveness ratio (ICER) for Policy B, Policy C and Policy D vs. Policy A.

**Results:**

At a willingness to pay (WTP) threshold of 3 times the national GDP per capita ($38,198), Policy B was not cost-effective compared to Policy A, with an ICER of $47,968 per QALY due to QALYs lost introduced by the delayed diagnosis of B12 deficiency and the potentially underestimated costs associated with treating neuropathy. However, Policy C and Policy D were cost-effective compared to Policy A, with an ICER of $32,615 and $20,287 per QALY, respectively. A probabilistic sensitivity analysis showed that there would be a 72.7% and 83.5% chance that the additional cost of Policy C and Policy D, compared with Policy A, was at or below the WTP threshold.

**Conclusions:**

Folate supplementation with integrated screening for B12 and folate deficiencies is considered the most cost-effective strategy for primary stroke prevention in hypertensive elderly patients in China. Future research should focus on advancing precision medicine to assess the feasibility and cost-effectiveness of nationwide implementation across diverse sub-populations within the context of integrated screening, ensuring efficient and tailored public nutrition strategy delivery.

**Supplementary Information:**

The online version contains supplementary material available at 10.1186/s12889-024-21005-7.

## Introduction

China faces a significant burden of stroke. According to the Global Burden of Disease Study 2019, stroke is the leading cause of disability-adjusted life years (DALYs) in China, increasing by 36.7% from 1990 to 2019 [1]. The economic impact is profound, with the weighted average annual medical cost per stroke patient being RMB10,637 (USD1,610), and an out-of-pocket cost of RMB3,093 (USD468). [2] Beyond the direct costs, stroke has severe implications for patient functioning, leading to long-term disability and dependency, which further stresses the public health system [3]. As the population ages and the prevalence of risk factors like hypertension remains high, the stroke burden is expected to rise, underscoring the urgent need for effective interventions to mitigate this growing public health challenge [4].

In China, it is crucial to acknowledge that the efficacy of folic acid in stroke prevention might be viewed within the context of a comprehensive strategy addressing multiple risk factors including hypertension, cholesterol levels, physical inactivity, etc. Lifestyle modification (i.e., diets, exercises, public health initiatives for smoking cessation and alcohol reduction, etc.) requires sustained public engagement and individual commitment. However, folic acid supplementation can be efficiently administered through food fortification, especially for those with low baseline dietary folate intake, reaching a broad population at a relatively low cost compared to pharmacological medications like antihypertensives and statins require ongoing medical management and come with higher direct costs.

Evidence from trials in China showed that folic acid supplementation was effective in stroke prevention for hypertensive adults [5, 6]. However, the beneficial effects of folic acid therapy in the primary prevention of stroke depend on the pre-existence of low folate status in the underlying population. China is known to have high rates of folate deficiency, with the prevalence rates ranging from 6.2% in southern regions and 38% in northern regions [7], in part because of the low folate content of China’s main staple crop, rice. Despite government initiatives to raise awareness about folic acid benefits, particularly for women of reproductive age and those at risk of cardiovascular diseases, there is no mandatory folate fortification of staple foods in China [8]. Conversely, although lacking mandatory fortification, India has advanced large-scale staple food fortification (LSFF) by integrating fortified wheat and rice into public distribution systems for specific states with low folate levels, aiming to address widespread micronutrient deficiencies [9]. In contrast, there was no benefit of folate in preventing stroke in a cohort of 3,600 patients from 55 clinical centers in the USA and Canada and 1 in Scotland, where the mean folate levels at baseline were about 28 nmol/L (12.4 ng/ml), 50% higher than in the China Stroke Primary Prevention Trial (CSPPT) at around 8.1 ng/ml [10].

Folate and vitamin B12 play a role in the body’s production of red blood cells. Deficiency of either can lead to anemia, where the red cells are larger than normal (macrocytosis). Unlike folate deficiency, untreated deficiency of vitamin B12 can also lead to permanent degeneration of the nerves in the spinal cord. Clinical practitioners do not routinely screen for folate deficiency, but laboratory tests for both vitamin B12 and folate levels are indicated when there are symptoms and signs of macrocytic anemia. While a deficiency of either vitamin B12 or folate can cause macrocytic anemia, giving folate to a patient with an underlying vitamin B12 deficiency can resolve the anemia but leave the processes of nerve degeneration unchecked. This is called the “masking” effect of folate. Patients whose B12 deficiency is masked by the use of folate can develop nerve degeneration that would have been prevented had their signs of anemia triggered testing for B12 deficiency. Earlier detection allows them to be treated before permanent nerve damage occurs [11].

**Fig. 1:**
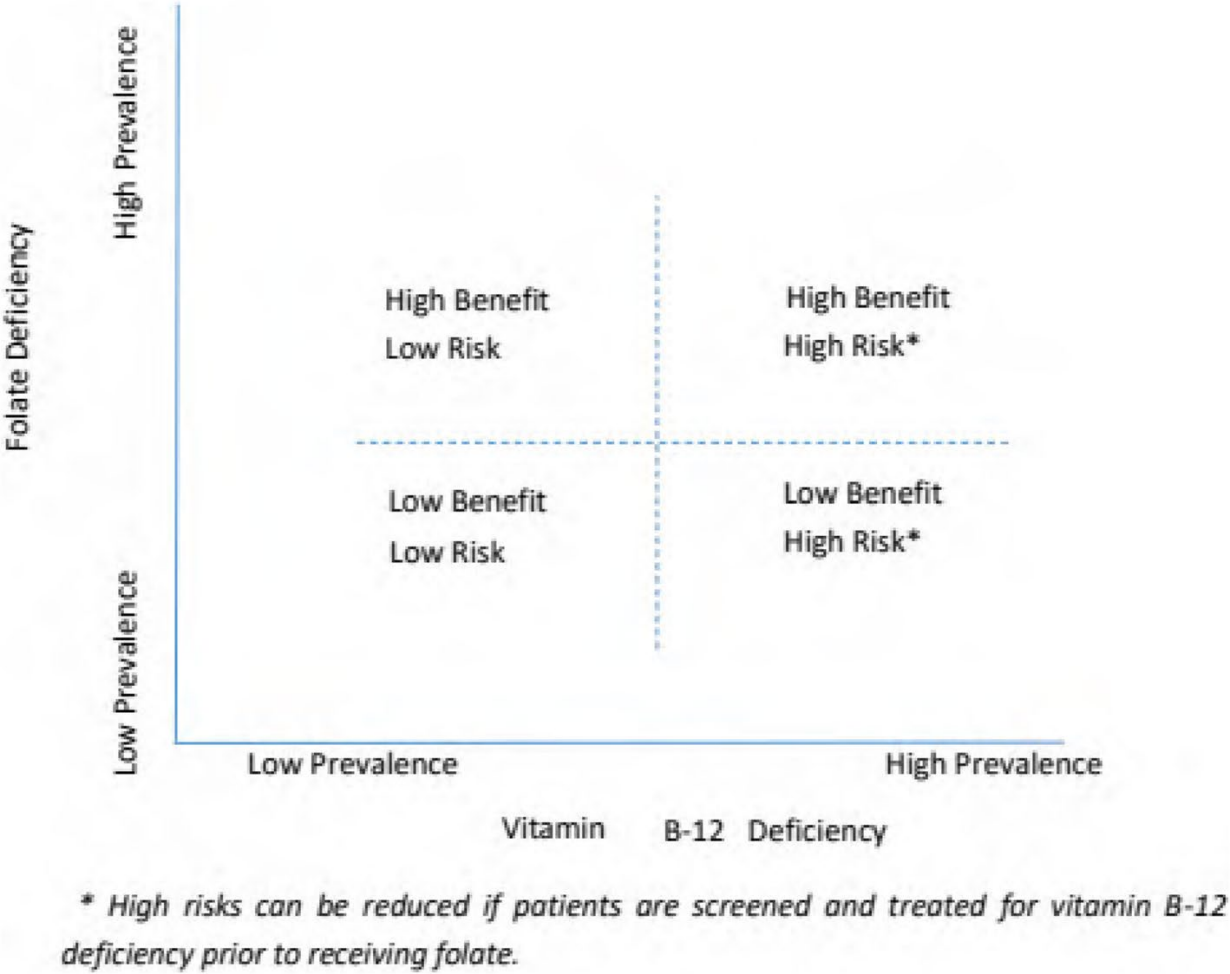
Benefits and risks of folate supplementation as a function of prevalence of folate and vitamin B12 deficiency

Other than B12 deficiency masking and rare patients with allergies, the use of folate supplements is safe. However, the problem of B12 deficiency masking creates a barrier to policies of routine folate supplementation or folate fortification of the food supply. The prevalence of B12 deficiency in China is around 14.1%, with seasonal variations [12]. The risks of folate supplementation, and hence the ratio of benefit to risk, will depend heavily on the prevalence of underlying vitamin B12 deficiency and folate deficiency. Figure 1 shows how the risks and benefits of a population-wide policy of folate supplementation correlate with the prevalence of folate and vitamin B12 deficiency. In settings where there is a high prevalence of both B12 and folate deficiency, such as China (lying in the first quadrant), the risks of folate supplementation can be mitigated by a policy of B12 screening prior to starting folate and treating patients with B12 deficiency with B12 supplements.

Meanwhile, even though Zhang et al. conducted a cost-effectiveness analysis evaluating the 5-year trial and lifetime cost-effectiveness based on the CSPPT [13], none of the previous studies have addressed the critical issue of vitamin B12 deficiency masking caused by folate supplementation in the context of primary stroke prevention in China. Our study, additionally, emphasizes evaluating cost-effectiveness from a nationwide nutrition policy implementation perspective, focusing on hypertensive individuals over 50, with the government as the payer. This policy-oriented and population-based approach enhances the practical relevance of our research, offering actionable insights for government decision-making.

Thus, against this backdrop, our study objective is to identify the most cost-effective policy intervention taking into account the cost of lab testing and the relative benefits and risks that depend on the prevalence of both vitamin B12 and folate deficiency. We will consider the following policy options:

A. Do nothing to address folate status in patients at risk for stroke. (Do Nothing)
B. Supplement with folate but no pre-screening for B12 deficiency. (Supp Only)
C. Supplement with folate, pre-screen all patients for B12 deficiency, and add B12 supplements if vitamin B12 is deficient. (Screen B12 & Supp)
D. Supplement with folate and B12 only for those who have folate deficiency, pre-screen all patients for both B12 and folate deficiencies. (Screen B12 and folate & Supp only patients with folate deficiency)

Policy A has the lowest intervention costs but has the least possibility of addressing folate-preventable strokes. Policy C has the highest costs due to the inclusion of laboratory tests, while Policy D, despite also requiring laboratory tests, has relatively lower costs as it only supplements patients with folate deficiency. The costs related to Policy C and D may or may not be worthwhile depending on the prevalence of vitamin B12 and folic acid deficiency.

## Methods

### Model structure

Our study specifically targeted hypertensive patients aged 50 and older in China, with around 0.3 billion population estimated by the China Population Census Yearbook 2022 and the prevalence study by Lloyd-Sherlock et al. [14, 15] This demographic was selected based on elevated stroke risk factors associated with hypertension and age, which aligns closely with the population studied in the CSPPT. This targeted focus enhances the applicability and relevance of our findings to real-world clinical and public health settings. A cost-effectiveness approach was used to assess the costs and QALY outcomes in the four groups: A. Do Nothing, B. Supp Only, C. Screen B12 & Supp, and D. Screen B12 and folate & Supp only folate deficiency. All groups in the model, including the do-nothing group, were assumed to be receiving appropriate treatment for their hypertension that was at least as good as the enalapril arm, which was the control group of the CSPPT study.

B12 deficiency symptoms typically manifest after 4 years of deficiency [16]. Generally speaking, the masking of vitamin B-12 deficiency is considered to become a risk at intakes > 1 mg/d [11, 17]. However, Savage and Lindenbaum's review of 38 B12 deficiency cases treated with less than 1 mg of folic acid found minimal anemia, but neurological deterioration (ostensibly due to masking) occurred in six patients, all of whom were treated for significantly longer durations (90–930 days) [18]. This highlights the importance of treatment duration in the risk of neurological complications. Given the information, we assumed that pernicious anemia would be masked in all individuals with B12 deficiency if folate was supplemented for four years in our model.

**Fig. 2:**
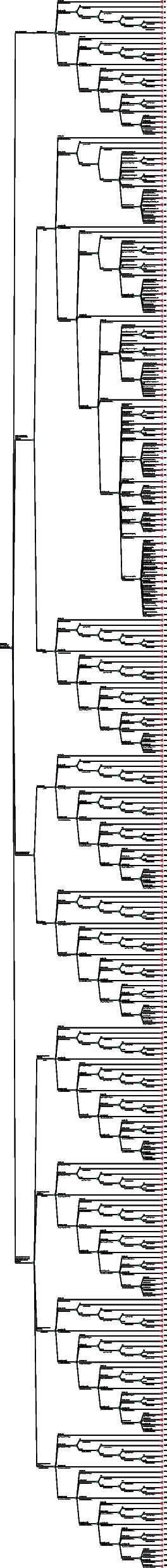
Decision Tree Model Depicting Four Policy Pathways in Primary Stroke Prevention

Figure 2 shows the decision tree model representing a five-year time horizon for the four groups. For each group, the decision tree incorporated a chance node for participants developing stroke or not developing stroke annually, and a subsequent chance node for death or survival. For Group A (Do Nothing), Group C (Screen B12 & supplement), and Group D (Screen B12 and folate & supplement only folate deficient), the risk that the treatment would not be associated with neuropathy due to folate-induced delayed diagnosis of vitamin B12 deficiency was set at zero. (Group A would not receive folate, so they could not be at risk that folate would induce masked vitamin B12 deficiency and Group C and D would be screened for vitamin B12 deficiency.) However, for Group B (Supp only), patients faced an annual risk of developing neuropathy at the beginning of the fourth year. We differentiate between the probabilities of developing severe, mild, and no neuropathy as a result of vitamin B12 deficiency. We have incorporated distinct probability estimates for each neuropathy state (severe neuropathy, mild neuropathy, and no neuropathy) to capture the variation in associated reductions in QALYs and are integrated into the cost estimates, where their death and survival situations were also traced. The incremental effect and costs were summarized using an incremental cost-effectiveness ratio (ICER) with reference to Policy A using formula 1.

1 $$\text{ICER}=\frac{({\text{C}}_{1}-{\text{C}}_{0})}{({\text{E}}_{1}-{\text{E}}_{0})}$$

The model components contained are shown in Table 1.

**Table 1:** The components contained in each group in the decision tree

Group	Year 1 to 5 Model Includes	Cumulative
A. Do Nothing	Intervention cost (Zero)	Costs QALYs
	Stroke costs	
	QALYs lost from stroke	
B. Supp Only	Intervention cost	Costs QALYs
	Stroke costs	
	Neuropathy costs^a^	
	QALYs lost from stroke	
	QALYs lost from Neuropathy	
C. Screen B12 & Supp	Intervention cost	Costs QALYs
	Stroke costs	
	QALYs lost from stroke	
D. Screen B12 and folate & Supp only folate deficiency	Intervention cost	Costs QALYs
	Stroke costs	
	QALYs lost from stroke	

^a^Assumed as zero in our model, see details in the policy implication part in Discussion section

Additionally, deterministic and probabilistic sensitivity analyses were conducted. One-way sensitivity analysis was done and then the tornado diagram regarding Policy C and Policy D vs. Policy A was drawn, which aims to evaluate changes in cost per QALY, thus demonstrating the effects of uncertainty on costs and health outcomes. The cost of an enalapril-folic acid separate pill and quality of life weight for stroke was used to conduct the threshold analysis to explore the maximum level of compensation required with screening intervention above which Policy C and D is not considered cost-effective. Moreover, probabilistic sensitivity analysis was performed to simulate all parameters’ changes simultaneously using Monte Carlo simulation with 1,000 replications for Policy C and Policy D vs. Policy A, and then the scatterplot of 1,000 iterations of Monte Carlo simulations and the cost-effectiveness probabilistic acceptance curve regarding Policy B, Policy C, and Policy D, and the alternative assumption scenario of using enalapril-folic acid combination pill (combo pill) under Policy C were also figured. The upper and lower bounds and their sources are listed in Table 2. The subgroup analysis by sex, age, smoking status, MTHFR C677T genotype, and homocysteine under Policy C was conducted.

**Table 2:** Probabilities, costs and utility parameters in the decision tree model

Parameter (Unit)	Base Case value	Lower Bound	Upper Bound	Source
**Annual Probability**				
Probability of B12 Deficiency (%)	0.141	0.139	0.145	[12]
Probability of Folate Deficiency (%)	0.273	0.232	0.318	[19]
Probability of Dual Deficiencies (%)	0.092	0.077	0.103	[20]
Hazard Ratio for First Stroke	0.79	0.68	0.93	[6]
Hazard Ratio for First Stroke for Folate-deficient Sub-population	0.61	0.45	0.82	[6]
Probability of First Stroke in the Control Group for Folate-indeficient Sub-population (%)	0.0061	\	\	[6]
Probability of First Stroke in the Control Group (%)	0.0070	\	\	[6]
Probability of First Stroke in the Treatment Group for Folate-deficient Sub-population (%)	0.0057	\	\	[6]
Probability of First Stroke in the Treatment Group (%)	0.0055	\	\	[6]
Probability of Death due to Stroke in the Treatment Group (%)	0.013	\	\	[6]
Probability of Death due to No-Stroke in the Treatment Group (%)	0.0056	\	\	[6]
Probability of Death due to Stroke in the Control Group (%)	0.0057	\	\	[6]
Probability of Death due to No-Stroke in the Control Group (%)	0.0061	\	\	[6]
Probability of Severe Neuropathy in case of B12 Deficiency	0.33	0.26	0.40	[18]
Probability of Neuropathy in case of B12 Deficiency	0.063	0.034	0.098	[18]
Probability of Death due to Neuropathy after Stroke	0.0073	0.0058	0.0093	[21]
Probability of Death due to Neuropathy after No-Stroke	0.0343	0.0303	0.0388	[21]
**Annual Costs**				
Cost of Enalapril & Folic Acid ($ Per day)				
Enalapril-folic acid separate pill	0.2417	0.0357	0.6126	[22]
Enalapril-folic acid combo pill	0.7123	0.7034	0.7584	[22]
Cost of Vitamin Folate Screening Test ($ Per visit)	9.3681	7.0261	11.7101	[23]
Cost of Vitamin B12 Screening Test ($ Per visit)	9.3681	7.0261	11.7101	[23]
Cost of Vitamin B12 ($ Per period)	11.6581	4.9963	16.6544	[22]
Direct Annual Medical Cost of a Stroke ($)	1,610.35	1,207.76	2,012.94	[2]
Effects				
Quality of Life for Stroke	0.719	0.560	0.879	[24]
Quality of Life for No-stroke	0.908	0.893	0.923	[24]
Quality of Life for Vitamin B12 Severe Neuropathy	0.310	0.170	0.450	[25]
Quality of Life for Vitamin B12 Neuropathy	0.588	0.200	0.920	[11]
Discount rate				
Annual Discount Rate	5%	0	8%	[26]

### Model data

Parameters are summarized in Table 2. The key data on folate supplementation effectiveness comes from the CSPPT study in China due to its largest cohort without a history of stroke and myocardial infarction, detailed data on individual baseline folate levels rather than ecological data, and robust study design [6]. In this study, a total of 20,702 hypertensive patients without a history of stroke from 32 communities in Jiangsu and Anhui provinces in China were randomized to enalapril-folic acid (*n* = 10,348) or enalapril alone (*n* = 10,354). The study participants had an average age of 60.0 years (SD, 7.5 years), and approximately 59% were female. The baseline median levels of folate and B12 are around 8.1 ng/ml and 379.7 pg/ml as shown in Table S3, with possible folate deficiency [27] rate (nearly < 5.6 ng/mL) rate accounting for 1.1% for the control group and 0.7% for the treatment group, and B12 deficiency (< 200 pg/mL) rate being responsible for 1.5% in both groups. During a five-year follow-up, a first stroke occurred in 282 participants (2.7%) in the enalapril–folic acid group compared with 355 participants (3.4%) in the enalapril group between 2008 and 2013, indicating an absolute risk reduction of 0.7% and a 21% relative risk reduction with a 95% CI of 0.68–0.93. Based on CSPPT data, each group's stroke and stroke-free probabilities, as well as the death and survival probabilities in each stroke and stroke-free branch were calculated, respectively, then converted from a five-year probability *P* into a one-year probability using formula 2.

2 $$Annual\;Probability=1-\text{exp}((\text{In}(1-P))/5)$$

Prevalence of vitamin B12 deficiency (Normal B12 status included VB12 levels > 258 pmol/L or VB12 levels ranging from 148 to 258 pmol/L with tHcy levels below 14 µmol/L) in China is 14.1%, retrieved from a comprehensive report across 14 provinces in China [12]. The prevalence varies slightly by season, ranging from 13.9% in winter and spring to 14.5% in summer and autumn. The nationwide prevalence of folate deficiency and dual deficiency are estimated to be 27.3% and 9.2%, respectively [19, 20]. The probability of developing severe neuropathy is 0.33, and the likelihood of any neuropathy is 0.063, based on a 17-year study in New York [18]. The probabilities of death due to neuropathy are 0.0073 after a stroke and 0.0343 without a stroke, derived from 5-year NHANES data by Hicks et al. [21] These probabilities were all converted to one-year probabilities using Formula 2.

The government-payer perspective for costing was used in our analysis. The reference year of the cost components and the GDP was 2022. Chinese RMB costs were converted to dollars using the average exchange rate for 2022 of 0.1487 USD per RMB [22]. Costs of folate-enalapril and vitamin B12 supplements were obtained from the 2022 median bidding price of local official Chinese documents [28]. Costs of stroke prevention in the treatment group were annualized by multiplying the wholesale cost of 0.8 mg of folic acid by 365 days to annualize the cost. The cost of vitamin B12 supplementation for individuals who required it was annualized based on a typical therapeutic dose of 500 mcg per day [29]. The costs of B12 and folate testing per unit were obtained from Chinese hospital official documents [23]. The outcome measure of benefit was QALYs gained per year. The utility weight for adults with vitamin B-12 deficiency was assumed to be 0.588 based on a study in 42 patients aged > 65 y with peripheral neuropathy using the HUI-3 (*n* = 42) [11]. The utility weights for participants with severe vitamin B12 deficiency masking-related neurological complications were assumed to be 0.31, estimated from Quality of Well-Being Index (QWB) scores in the Reference Case analysis [25]. The utility weight for stroke and hypertensive participants without stroke and neuropathy resulting from vitamin B12 deficiency were both assumed to be 0.719 and 0.908 according to Xie et al. [24]

A five-year period was used as the time horizon since the CSPPT clinical trial program impacted healthcare costs and benefits over this period. For each year, costs and benefits were discounted using the annual discount rate of 5% according to Chinese Guidelines for Pharmacoeconomic Evaluations (2020 Editions) [26]. The annual direct cost of stroke, converted to USD, was used to multiply the stroke life-years accrued under Policy A as a reference group. A similar approach estimated the medical costs of stroke for Policy B and Policy C, respectively to capture potential cost offsets from stroke prevention. Intervention costs were estimated from the Chinese healthcare sector and focused solely on the costs of supplementation and screening. The patient’s time and travel costs were assumed to be negligible.

## Results

### Base case results

The results for the base scenario are presented in Table 3. In terms of Policy A, the expected medical costs of intervention were predicted to be $140.59 per patient. In contrast, the expected value of the medical costs of stroke in Group A was projected to be $138.19, which was higher than the expected medical costs of a stroke in Group B, C and D. As expected, Group A had prevented the fewest strokes because it was a reference group where no folic acid supplementation in place. Meanwhile, the total expected QALYs were predicted to be 3.8699.

**Table 3:** Results of the base case cost-effectiveness analysis

Components	Group A Do Nothing	Group B Supp only	Group C Screen (B12) & Supp	Group D Screen (Folate and B12) & Supp (Folate Deficient)
Expected medical costs of intervention	US$140.59	US$377.84	US$388.33	US$224.77
Expected medical costs of stroke	US$138.19	US$108.08	US$108.08	US$118.38
**Total medical costs**	US$278.78	US$485.92	US$496.40	US$343.15
**Total expected QALYS**	3.8699	3.8742	3.8766	3.8731
**Incremental cost vs. A**		207.13	217.62	64.37
**Incremental QALYs vs. A**		0.0043	0.0067	0.0032
**ICER with respect to Group A**		47,967.75	32,614.71	20,286.94

**Fig. 3:**
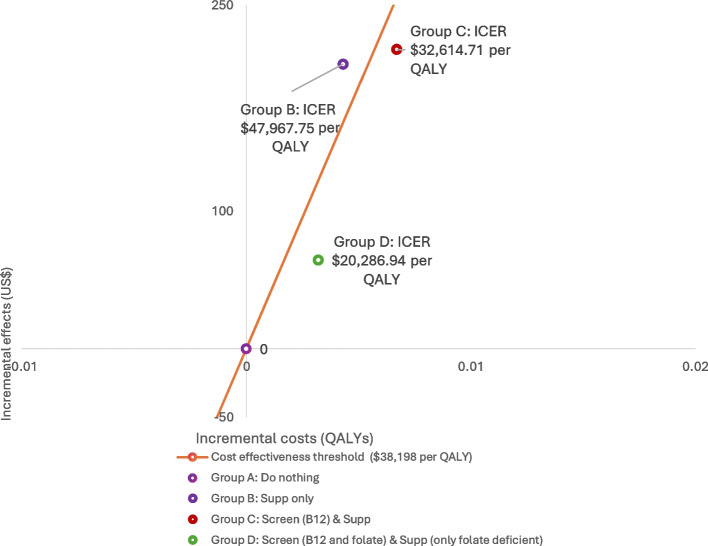
Cost-effectiveness plane for Policy C (Screen & Supp) vs. Policy A (Do Nothing)

Over the five years, the incremental cost and QALYs gained by Group B vs. Group A were estimated to be $207.13 and 0.0043, respectively. Overall, the ICER of Policy B with respect to Policy A was $47,968 per QALY. Policy B is unacceptable because it raises costs and lowers health, considering the underestimation of neuropathy costs associated with the delayed diagnosis of B12 deficiency-related neuropathy due to data unavailability and thus the impacts on ICER might be anti-conservative (i.e. favoring folate supplementation). (Figure 3) In Policy C, the incremental costs and QALYs gained by Group C vs. Group A were estimated to be $217.62 and 0.0067, respectively, and finally, the ICER of Policy C vs. Policy A was $32,615 per QALY, indicating that Policy C would be cost-effective under a willingness to pay (WTP) threshold of 3 times the national GDP per capita ($38,198; 256,877 RMB). Policy C would lie in the right upper quadrant of the cost-effectiveness plane. (Figure 3) Under Policy D, the ICER was estimated as $20,287 per QALY, with incremental costs and benefits of $64.37 and 0.0032, respectively. Policy D incurred relatively lower costs as folate supplementation would be limited to patients with folate deficiency, despite the additional screening costs for both B12 and folate. However, Policy B and C generated more QALYs than Policy D because they provided folate benefits to a broader population, including those with homocysteine deficiency or genetic abnormalities, who did not receive these benefits under Policy D. This Policy D would be most cost-effective under the WTP threshold (Fig. 3).

### Sensitivity analysis results

The results for the one-way sensitivity analysis of Policy C and Policy D vs. Policy A are presented in SFigure 1 and SFigure 2 in Supplementary Material, respectively. For Policy C, the results tend to be entirely sensitive to the hazard ratio for the first stroke, cost of enalapril-folic acid drug combinations, and quality of life weight for stroke, which implied that it would not be cost-effective anymore if the HR > 0.83, or cost of enalapril-folic acid pill was greater than $0.27 per day or quality of life weight for stroke was greater than 0.77 adjusting for other variables. For Policy D, it would not be cost-effective anymore if the HR > 0.85, or the cost of the enalapril-folic acid pill was greater than $0.38 per day or the quality of life weight for stroke was greater than 0.84 adjusting for other variables.

The probabilistic sensitivity analysis indicated that there would be a 72.7% and 83.5% chance that the additional cost of Policy C and Policy D, compared with Policy A, is at or below 3 times the GDP per capita threshold as shown in SFigure 3 and 4 in Supplementary Material. The cost-effectiveness probabilistic acceptance curves are depicted in SFigure 5 in Supplementary Material. Additionally, the probability of enalapril-folic acid being cost-effective based on a threshold of 3 times the GDP per capita fell to 52.5% under Policy B (Supp only) and rapidly fell to 0 under the assumption that we treat hypertensive patients with daily combo pills.

### Subgroup analysis results

Subgroup analysis results of Policy C vs. Policy A are summarized in Table S1 in Supplementary Information, where Table S2 presents the hazard ratio for the first stroke for each subgroup. The ICER for males was $19,854 per QALY gained, with a 69.0% probability of being cost-effective at a threshold of 3 times the GDP per QALY. In contrast, for females, the ICER was significantly higher at $50,622 per QALY, with only a 59.0% probability of being cost-effective at that threshold. Age-wise, individuals aged 55 to 64 exhibited a lower ICER of $24,179 per QALY and a 77.9% probability of cost-effectiveness, outperforming both younger (under 55 years, $50,199 per QALY) and older (over 65 years, $36,475 per QALY) cohorts. Among the MTHFR genotypes, the probability of cost-effectiveness at the threshold was 56.9% for CT, 85.1% for CC, and 76.0% for TT. For homocysteine levels, patients with levels < = 10.5 μmol/L had a 58.9% probability, while those with levels above 10.5 μmol/L had about a 70% probability of being below the threshold.

## Discussion

To our knowledge, this study is the first to provide a comprehensive cost-effectiveness analysis of folate supplementation for primary stroke prevention, with a critical consideration that higher folate intake could dangerously delay the diagnosis of underlying vitamin B12 deficiency, potentially leading to irreversible neurological damage. To resolve it, our model indicates that the integrated screening strategy before folate supplementation (Policy D) would be the most cost-effective, which could be integrated into Chinese nationwide nutrition fortification strategies to mitigate potential adverse effects caused by folic acid supplements. This policy-oriented and population-based study pioneers a strategic framework for optimizing stroke prevention decision science.

Policy A was doing nothing to address folate status in patients at risk for stroke, which was set as the reference group and had the lowest intervention costs but had the lowest benefits because it did nothing to address folate-preventable strokes. Policy B was supplementing with folate, but it imposed no pre-screening for B12 deficiency. Policy B was not cost-effective because of health harms from neuropathy due to delayed diagnosis of vitamin B12 deficiency because folate treatment would mask the anemia.

Policy C refers to supplementing with folate, pre-screening all patients for B12 deficiency, and adding vitamin B12 supplements if vitamin B12 is deficient. Policy D was supplementing with folate only for patients with folate deficiency, pre-screening all patients for B12 deficiency and B12, and adding vitamin B12 supplements if vitamin B12 is deficient. With Policy C and D, QALYs gained could potentially justify the higher intervention costs due to the addition of laboratory tests and B12 supplements. Notably, Policy D could be regarded as the most cost-effective for the hypertensive population over age 50 in China, given the high prevalence of underlying folate and B12 deficiency in this population.

Over a lifetime, under Policy D, the cumulative benefits of reduced stroke incidence due to folate fortification and enhanced quality of life gained from preventing people from a delayed diagnosis of neuropathy due to masking of anemia could significantly outweigh the initial costs associated with folate supplementation, B12 and folate deficiency test, and B12 treatment. Thus, our current 5-year horizon may not capture these extended benefits, thus potentially underestimating the full economic value of the intervention. However, under Policy B, the QALYs lost due to delayed diagnosis of neuropathy would be underestimated over the 5 years, since some literature suggested that this kind of neuropathy might be irreversible in some cases.

A previous cost-effectiveness analysis of folic acid supplements in primary stroke prevention by Zhang et al. was informative and valuable, estimating that the ICER of enalapril-folic acid, compared to enalapril alone, was $44,127.13 (284,620.00 RMB) per QALY gained and $26,066.13 (168,126.54 RMB) per QALY gained, over a 5-year in-trial period and over a lifetime horizon, respectively [13]. This prior estimate’s ICER is similar to the ICER of Policy B vs. Policy A in our study. Our slightly higher ICER is due to modeling and parameterization differences. Unlike the prior study, we took note of the potential harm from masking vitamin B12 deficiency.

Specifically, in our model of Policy B, the costs of vitamin B12 screening and treatment were included when we predicted the total medical costs. Furthermore, our model excludes patients found to be B12 deficient from folate treatment which lowers the number of patients who benefit from Policy C as a means to prevent stroke. Meanwhile, unlike the Zhang et al. (2022) study our study did not consider multiple strokes and did not include branches that modeled the incidence of cardiovascular diseases. We chose not to model cardiovascular disease events because all beneficial effects of folate acid in CVD prevention were not statistically significant in the CSPPT [6]. Another important difference in our cost parameters compared to Zhang et al. (2022) was that our cost model used estimated the medication costs by having patients take an enalapril pill and folic acid pill separately (separate pill) for the daily treatment rather than the substantial more expensive combo pill. The separate pills are both generic. It seemed arbitrary to insist that patients and the providers giving helpful medical advice would choose to pay an extra $0.47 for the luxury of a combo pill when the separate pills would be equally effective. However, out of curiosity we also simulated the alternative scenario that everybody would take the combo enalapril-folate pill in SFigure 5. The alternative model of Policy C indicated that with combo pills would generally not be cost-effective, aligning well with the findings from Zhang et al. whose model was based on the cost of combo pills [13]. It is worth mentioning that using separate pills for enalapril and folic acid instead of a combination pill could be considered a limitation of the study, despite the cost savings. The separate pills may introduce a higher pill burden, which could negatively impact patient adherence. Lower adherence could reduce the effectiveness of the treatment, potentially offsetting the cost benefits. Addressing this issue would require patient education and possibly the use of reminder systems or packaging solutions, such as blister packaging, to simplify the regimen and encourage consistent medication use.

There are also limitations to the study. Firstly, the only adverse effect of folate supplements included in the model was the masking of vitamin B12 resulting in anemia and neurological impairments. However, other rare side effects like allergies might be caused by folic acid supplements, which indicates that the costs might be underestimated. Secondly, this study did not consider the participants with other co-morbidities except stroke, and vitamin B12 and folate deficiency. The presence of co-morbidities might lead our model to underestimate or overestimate QALYs gained by folic acid supplements. However, the CSPPT trial did not show any statistically significant benefits in terms of preventing cardiovascular disease with folate supplementation. Thirdly, this study did not consider the administrative costs of insurance and outreach to persuade providers and patients to choose the Policy C and D intervention and this might omit some true costs of the intervention. Meanwhile, a notable limitation of our study was the use of the three times GDP per capita as the threshold for willingness-to-pay (WTP). This threshold has been increasingly questioned by global health authorities, including the WHO, due to its potential to misalign with the economic realities and health priorities of different countries. Besides, the compliance rates observed in the CSPPT, with treatment discontinuation rates around 14% and a follow-up dropout of around 0.3% for both study groups, were maintained within a controlled clinical trial environment. This presents a limitation when extrapolating to real-world settings where compliance is often influenced by diverse factors such as patient education, access to healthcare, and socioeconomic variables. Lastly, the lack of hazard ratio for the first stroke by subgroup by folate level in the original trial data prevented us from conducting specific subgroup analyses for Policy D.

Accordingly, there are several policy implications. Firstly, our contribution adds to growing evidence that folate supplementation would be safe and cost-effective only if combined with pre-screening for vitamin B12 deficiency or integrated screening of both B12 and folate deficiency. Additionally, we emphasize the need for a context-specific approach to determining WTP thresholds that more accurately reflect the economic realities and health investment capacities of individual countries. Furthermore, in our analysis, we did not include the medical costs of B12 deficiency-related neuropathy in Policy B due to data limitations and the complexity of estimating these costs accurately, particularly within the Chinese context. This conservative approach likely underestimates the true economic costs under Policy B. Consequently, the cost-effectiveness of Policy B relative to Policy A may also be conservatively estimated. Acknowledging this underscores the need for more precise cost-of-illness data to better inform future cost-effectiveness. Notably, the subgroup analysis under Policy D might be of great importance to be supplemented for tailored intervention before policy implementation due to precision medicine.

To effectively implement Policy D within the framework of China's current healthcare practices, the policy could be integrated into the existing National Nutritional Improvement Program, which already focuses on micronutrient supplementation. This could be achieved by expanding routine B12 and folate screening within community health centers and linking it with ongoing folic acid supplementation programs targeting at-risk populations, such as pregnant women and the elderly. Additionally, leveraging the existing infrastructure for public health education could ensure widespread awareness and compliance, aligning with China’s focus on preventive healthcare.

## Conclusion

Folate supplementation is cost-effective for stroke prevention only if accompanied by initial screening for B12 deficiency or both B12 and folate deficiency for the hypertensive population older than 50 years old in China. The integrated screening for both B12 and folate deficiencies before folate supplementation would be considered the most cost-effective strategy, which needs to be recommended to the Chinese government. In contrast, the use of folate supplements without vitamin B12 and folate screening was not cost-effective due to the QALYs lost resulting from the delayed diagnosis of B12 deficiency and the potential costs associated with treating neuropathy that were not fully accounted for in our analysis. In summary, our model shows that integrated screening policies can control the side effects when folate and B12 deficiency are prevalent and can make the folate supplementation intervention cost-effective. Future research should further explore the feasibility and cost-effectiveness of nationwide implementation for diverse sub-populations within the context of integrated screening. This will be crucial for optimizing stroke prevention strategies and public nutrition policy delivery in China.

## Supplementary Information

Supplementary Material 1.

## Data Availability

No datasets were generated or analysed during the current study.
