# Supplementary material for: Revisiting cost-effectiveness of folic acid supplementation in primary stroke prevention in China: considering vitamin B12 deficiency masking issue
Source: BMC Public Health. 2024 Dec 19;24:3540. doi: 10.1186/s12889-024-21005-7 (PMC11661315; doi:10.1186/s12889-024-21005-7)
Supplement: Supplementary file 1 — Supplementary Material 1. [file 12889_2024_21005_MOESM1_ESM.pdf]

**SFigure 1. Tornado diagram of Policy C (Screen for B12 & Supply) vs. Policy A (Do Nothing)**

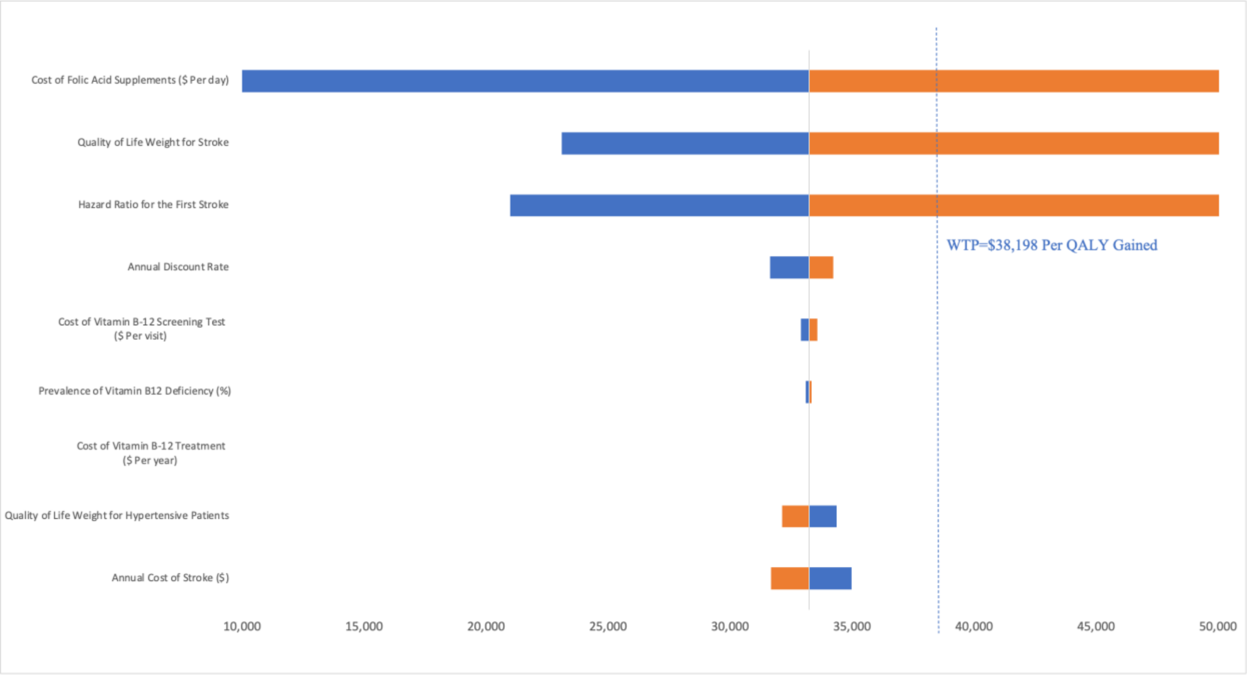

## SFigure 2. Tornado diagram of Policy D (Screen for B12 and folate & Supply for only folate deficient) vs. Policy A (Do Nothing)

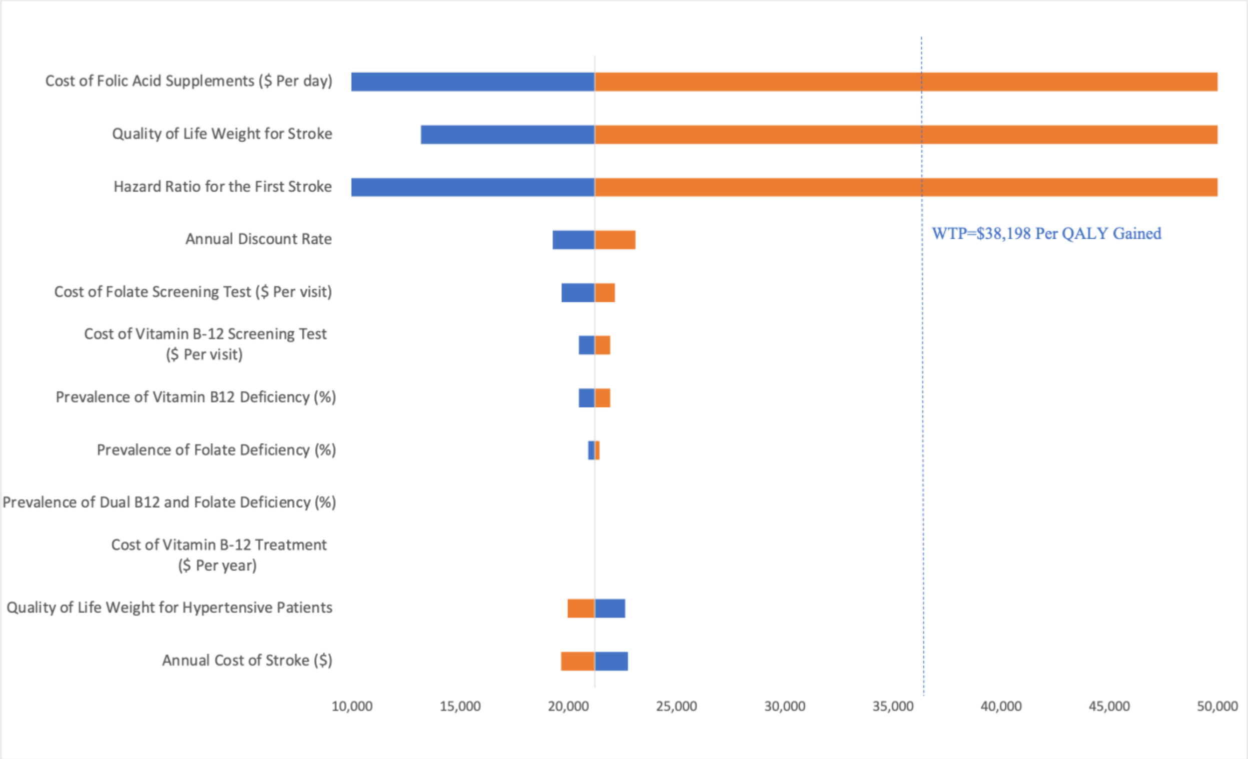

**SFigure 3. Scatterplot of 1000 Iterations of Monte Carlo Simulations for Cost-effectiveness plane for Policy C (Screen for B12 & Supply) vs. Policy A (Do Nothing)**

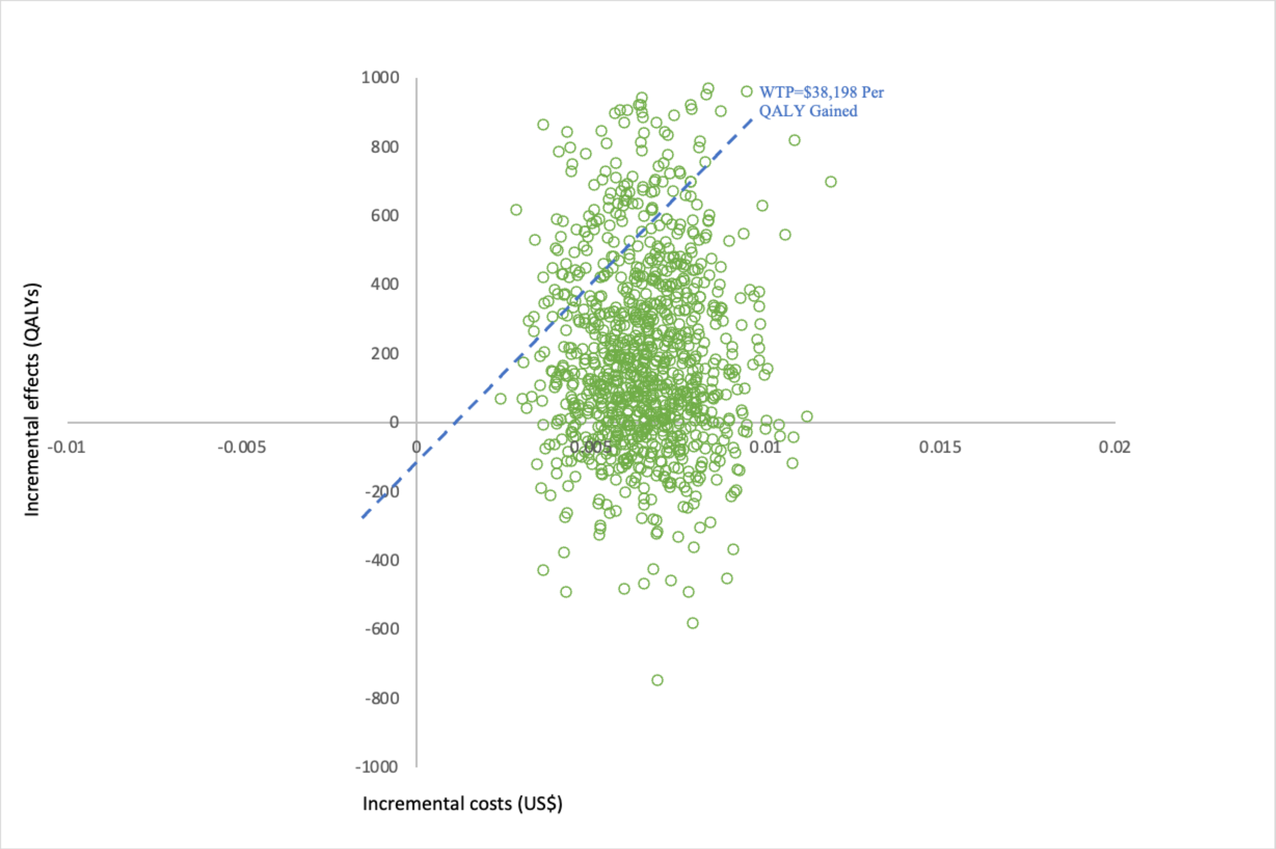

**SFigure 4. Scatterplot of 1000 Iterations of Monte Carlo Simulations for Cost-effectiveness plane for Policy D (Screen for B12 and folate & Supply for only folate deficient) vs. Policy A (Do Nothing)**

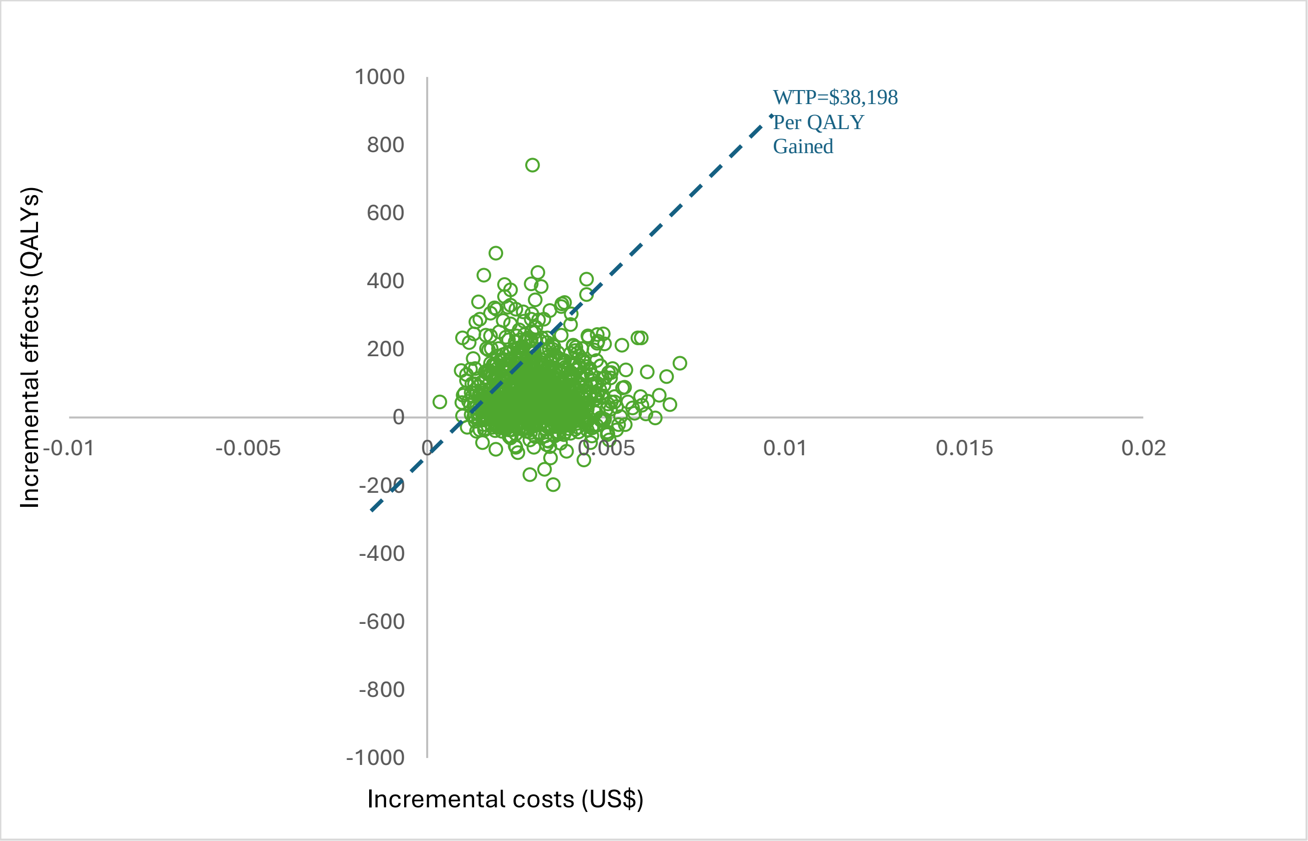

# SFigure 5. Cost-effectiveness Probabilistic Acceptance Curve

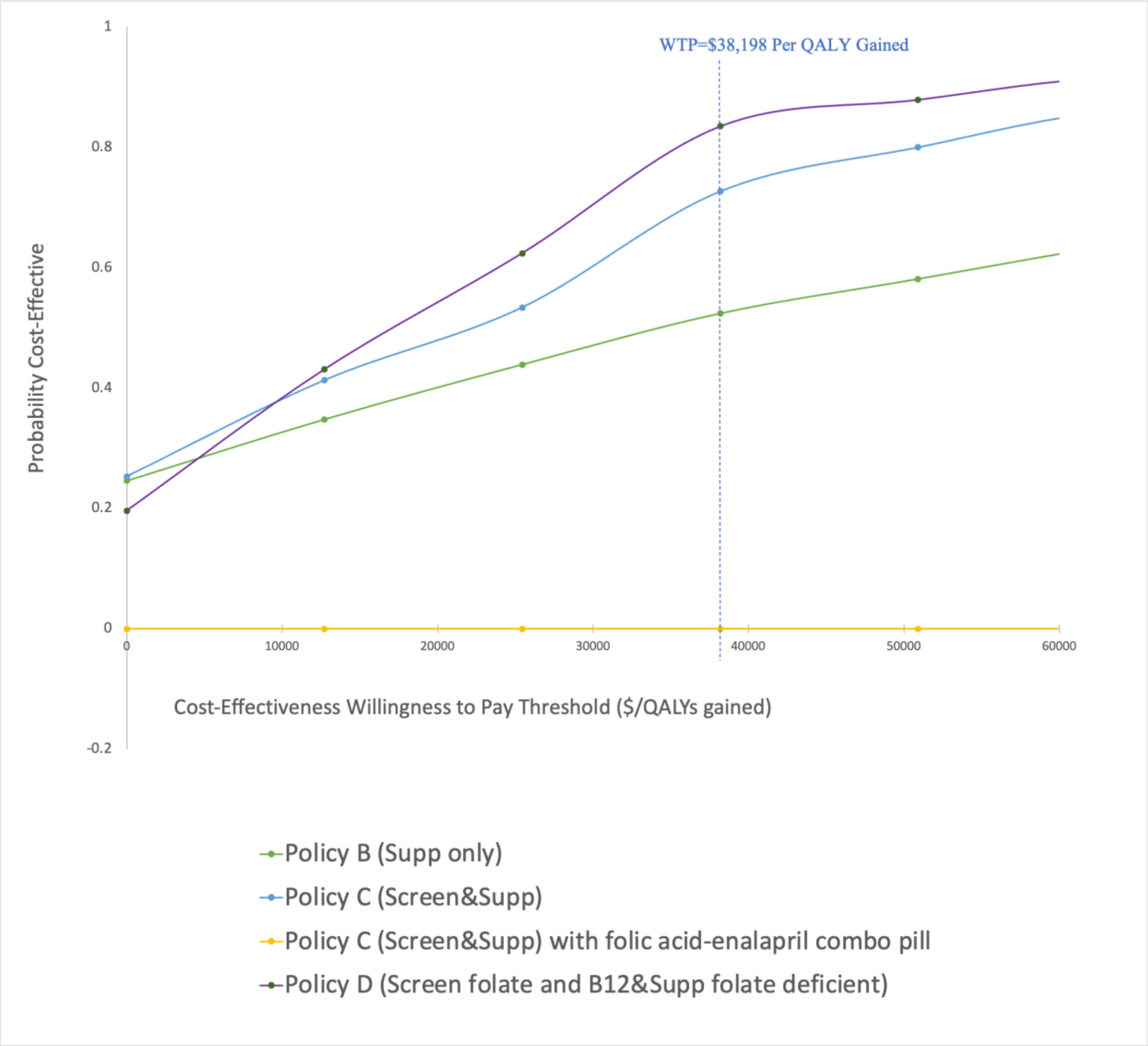

# Table S1. Cost-effectiveness for Policy C (Screen for B12 & Supply) vs. Policy A (Do Nothing) results for subgroups

| Subgroup | Cost ($) | | | QALYs | | | ICER | %<1 time of GDP/capita | %<2 times of GDP/capital | %<3 times of GDP/capita |
|---|---|---|---|---|---|---|---|---|---|---|
| | Policy C | Policy A | Δ | Policy C | Policy A | Δ | ($/QALY) | per QALY | per QALY | per QALY |
| **Sex** | | | | | | | | | | |
| Male | $503.26 | $311.49 | $191.77 | 3.8757 | 3.8661 | 0.0097 | $19,854.15 | 40.10% | 52.10% | 69.30% |
| Female | $491.63 | $256.46 | $235.17 | 3.8772 | 3.8725 | 0.0046 | $50,621.61 | 32.20% | 43.00% | 59.00% |
| **Age, yrs** | | | | | | | | | | |
| <55 | $460.16 | $223.91 | $236.25 | 3.8811 | 3.8763 | 0.0047 | $50,199.00 | 37.60% | 46.30% | 60.80% |
| ≥55 - <65 | $488.65 | $285.43 | $203.22 | 3.8775 | 3.8691 | 0.0084 | $24,179.03 | 43.10% | 58.10% | 77.90% |
| ≥65 | $543.96 | $323.41 | $220.56 | 3.8707 | 3.8647 | 0.0060 | $36,475.03 | 30.10% | 41.00% | 57.80% |
| **Smoking status** | | | | | | | | | | |
| Never | $482.43 | $262.44 | $219.99 | 3.8783 | 3.8718 | 0.0065 | $33,961.53 | 39.60% | 54.30% | 71.60% |
| Former | $523.94 | $320.96 | $202.99 | 3.8732 | 3.8650 | 0.0082 | $24,685.28 | 45.10% | 56.20% | 70.70% |
| Current | $527.05 | $311.51 | $215.54 | 3.8728 | 3.8661 | 0.0067 | $32,003.16 | 39.20% | 51.80% | 68.60% |
| ***MTHFR* C677T genotype** | | | | | | | | | | |
| *MTHFR* CC | $479.66 | $282.62 | $197.04 | 3.8787 | 3.8695 | 0.0092 | $21,461.00 | 53.60% | 67.40% | 85.10% |
| *MTHFR* CT | $501.21 | $263.11 | $238.10 | 3.8760 | 3.8718 | 0.0042 | $56,072.62 | 31.90% | 42.20% | 56.90% |
| *MTHFR* TT | $505.77 | $305.47 | $200.30 | 3.8754 | 3.8668 | 0.0086 | $23,168.72 | 45.60% | 59.40% | 76.00% |
| **Homocysteine, µmol/L** | | | | | | | | | | |
| ≤10.5 | $473.07 | $237.79 | $235.28 | 3.8795 | 3.8747 | 0.0047 | $49,608.67 | 33.10% | 42.20% | 58.90% |
| 10.5 - <12/5 | $483.97 | $264.43 | $219.54 | 3.8781 | 3.8716 | 0.0065 | $33,660.97 | 42.40% | 53.60% | 72.20% |
| 12.5 - <15.5 | $499.90 | $299.43 | $200.47 | 3.8762 | 3.8675 | 0.0087 | $23,148.41 | 45.80% | 60.40% | 78.10% |
| >=15.5 | $520.31 | $309.41 | $210.90 | 3.8736 | 3.8663 | 0.0073 | $28,820.55 | 42.90% | 53.30% | 68.70% |

## Table S2. Hazard ratios and 95% CI for the first stroke for subgroup analysis

| Variable | HR | 95% CI | |
|---|---|---|---|
| **Sex** | | | |
| Male | 0.68 | 0.54 | 0.86 |
| Female | 0.90 | 0.73 | 1.12 |
| **Age, yrs** | | | |
| <55 | 0.87 | 0.60 | 1.26 |
| 55 -65 | 0.70 | 0.55 | 0.89 |
| ≥65 | 0.86 | 0.67 | 1.10 |
| **Smoking status** | | | |
| Never | 0.79 | 0.64 | 0.96 |
| Current | 0.80 | 0.60 | 1.06 |
| Former | 0.76 | 0.60 | 1.26 |
| ***MTHFR* C677T genotype** | | | |
| *MTHFR* CC | 0.65 | 0.48 | 0.89 |
| *MTHFR* CT | 0.93 | 0.74 | 1.16 |
| *MTHFR* TT | 0.72 | 0.53 | 0.97 |
| **Homocysteine, µmol/L** | | | |
| ≤10.5 | 0.88 | 0.61 | 1.26 |
| 10.5 - <12/5 | 0.78 | 0.56 | 1.10 |
| 12.5 - <15.5 | 0.71 | 0.53 | 0.96 |
| >=15.5 | 0.79 | 0.59 | 1.05 |

## Table S3. Baseline characteristics of the study participants

| Characteristics | Enalapril-folic acid group (n=10348) | Enalapril group (n=10354) |
|---|---|---|
| Age, yrs (SD) | 60.0 (7.5) | 60.0 (7.6) |
| Male, n (%) | 4245 (41.0) | 4252 (41.1) |
| Body mass index, kg/m2 (SD) | 25.0 (3.7) | 24.9 (3.7) |
| Methylenetetrahydrofolate Reductase (MTHFR) C677T genotype, n (%) | | |
| CC | 2821 (27.3) | 2831 (27.3) |
| CT | 5095 (49.2) | 5081 (49.1) |
| TT | 2432 (23.5) | 2442 (23.6) |
| Smoking status, n (%) | | |
| Never | 7119 (68.8) | 7135 (68.9) |
| Former | 761 (7.4) | 809 (7.8) |
| Current | 2461 (23.8) | 2408 (23.3) |
| Baseline Systolic Blood Pressure, mm Hg (SD) | 166.8 (20.4) | 166.9 (20.4) |
| Mean Systolic Blood Pressure during treatment, mm Hg (SD) | 139.7 (11.1) | 139.8 (11.3) |
| Total cholesterol, mg/dL (SD) | 213.6 (46.0) | 213.2 (45.8) |
| High-Density Lipoprotein Cholesterol, mg/dL (SD) | 52.0 (14.0) | 51.8 (13.9) |
| Fasting glucose, mg/dL (SD) | 104.5 (30.6) | 104.5 (30.6) |
| Baseline folate, ng/mL (SD) | 8.5 (4.0) | 8.5 (4.0) |
| Homocysteine, µmol/L (IQR) | 12.5 (10.5 - 15.5) | 12.5 (10.5 - 15.5) |
| Vitamin B12, pg/mL (IQR) | 379.6 (314.3 - 475.2) | 379.6 (315.7 - 478.2) |
